# Alfalfa Silage Treated With Sucrose Has an Improved Feed Quality and More Beneficial Bacterial Communities

**DOI:** 10.3389/fmicb.2021.670165

**Published:** 2021-10-13

**Authors:** Jinhe Kang, Shaoxun Tang, Rongzhen Zhong, Zhiliang Tan, Duanqin Wu

**Affiliations:** ^1^CAS Key Laboratory for Agro-Ecological Processes in Subtropical Region, National Engineering Laboratory for Pollution Control and Waste Utilization in Livestock and Poultry Production, Hunan Provincial Key Laboratory of Animal Nutrition & Physiology and Metabolism, South-Central Experimental Station of Animal Nutrition and Feed Science in Ministry of Agriculture, Institute of Subtropical Agriculture, Chinese Academy of Sciences, Changsha, China; ^2^Northeast Institute of Geography and Agroecology, Chinese Academy of Sciences, Changchun, China; ^3^Institute of Bast Fiber Crop, Chinese Academy of Agricultural Sciences, Changsha, China

**Keywords:** alfalfa, silage, additive, bacterial community, fermentation quality

## Abstract

Alfalfa silage is one of the main roughages in the production of dairy cow, which can provide nutrition with high quality to improve milk quality and production. Sucrose additions have been widely used to improve the silage quality. In this study, the effects of sucrose on the fermentation quality and bacterial communities of alfalfa silage were investigated here using 0, 0.5, and 1% sucrose ensiling treatments for 15, 30, and 60 days. The ensiling time significantly decreased the crude fiber content and increased the ammonia nitrogen, acetic acid content, and the relative abundance of *Enterococcus* in the silages. The 1% sucrose-treated silage at 60 days had the lowest neutral detergent fiber acid, acid detergent fiber, and crude fiber content and the highest relative feed value. Moreover, sucrose-treated silage contained less acetic acid, propionic acid, and butyric acid, and had a lower pH than the controls for each duration. *Enterobacteriaceae*, *Klebsiella*, and *Enterococcus* were the dominant genera in all groups, and the relative abundance of *Enterococcus* and *Lactobacillus* was higher in the 1% sucrose-treated group than in the control. These results suggested that sucrose supplementation could improve alfalfa silage quality and increase its beneficial bacterial content.

## Introduction

Alfalfa is a perennial legume plant with a high nutritional quality and good palatability, and has been widely planted in China (Sengul and Sengul, 2008; Xu et al., 2020). However, alfalfa harvests are seasonal, and it is difficult to preserve fresh alfalfa for a long time due to its high water content, especially during the rainy season in southern China. Consequently, alfalfa is normally ensiled for use as an animal fodder (Li et al., 2016).

Alfalfa silage is one of the main roughages in the production of dairy cow, which can provide nutrition with high quality to improve milk quality and production. As a result, many practices have been applied to enhance the fermentation quality of alfalfa silage (Kung et al., 2003; Muck et al., 2018), including the supplementation of different sugar sources such as fructose, molasses, and pectin (Zhang et al., 2018; Wang et al., 2020). Sucrose additions have been widely used to improve the silage quality (Heinritz et al., 2012; Sun et al., 2020) because sucrose can increase the supply of substrates for the growth of lactic acid bacteria (Heinritz et al., 2012). Some *lactobacilli* can utilize citrate for the conversion of sugars into lactic acid and volatile fatty acids (de Figueroa et al., 2000). However, *Lactobacillus plantarum* addition is not favored in practical processing due to its difficulty in conservation and high price.

When ensiling forage, the types of microorganism that are present during fermentation also play a critical role (Kung et al., 2018). Thus, monitoring the ensiling process with respect to the changes in the chemical and microbial compositions could also help to improve our understanding of it (Namihira et al., 2010; Kung et al., 2018). Previous studies analyzed silage bacteria profiles to identify the ensiling processes using 16S rRNA sequencing (Ali et al., 2020). This experiment was carried out to further explore the effects of sucrose supplementation alone on ensiling characteristics and bacterial community compositions of alfalfa silage. The results of this study should provide theoretical support and guidance for future alfalfa silage production.

## Materials and Methods

### Forage Harvesting and Silage Preparation

Alfalfa (*Medicago sativa* L.) was mechanically harvested at the early bloom stage in May 2019 from an experimental field located in Yuanjiang City, Hunan Province, China. A commercial sucrose (Guangzhou HUATANG Co., Ltd., Guangzhou, China) was used as additive and mixed with alfalfa before ensiling. The alfalfa stocks were individually cut into pieces that were approximately 2 cm in length and then mixed and randomly assigned to three groups: AL group (the control with no sucrose additions), AS1 group (with 0.5% sucrose additions), and AS2 group (with 1.0% sucrose additions). Thirty-six vacuum plastic bags (25 × 40 cm; Zhengzhou Non-gshengle livestock equipment Co., Ltd., Zhengzhou, China) were used for the alfalfa ensiling (10.0 kg/bag) and sealed by a vacuum sealer (Airmate Electric Co., Ltd., Shenzhen, China). The silage was maintained in the bags for durations of 15, 30, and 60 days, respectively. Three bags were randomly selected for each sucrose treatment at each time point. Silage samples were then transferred from the bags to plastic boxes *via* an opening for homogeneous mixing and then used for chemical and microbial analyses.

### Chemical Analyses

The pre-ensiled alfalfa and silage samples were dried at 60°C for 48 h to a constant weight. Dry matter (DM) and crude protein (CP) levels were analyzed according to AOAC standards (AOAC International, 1995). Acid detergent fiber (ADF) and neutral detergent fiber (NDF) content was assessed using the methods described by Vansoest et al. (1991) by using a Fibretherm Fiber Analyzer (Gerhardt, Bonn, Germany). The gross energy (GE) was determined according to Bomb calorimeter method described by the International Organization for Standardization (ISO) 9,831–1,998 and by using isothermal auto-calorimeter (5E-AC8018, Changsha Kaide Measurement and Control Instrument Co., Ltd., China).

Silage sample (20 g) was homogenized with distilled water (180 ml) and then filtered through four layers of cheesecloth and a qualitative filter paper. The pH of the water extract was measured using a pH meter (Model PHSJ-4F, Shanghai Precision & Scientific Instrument Co., Ltd., China). Ammonia nitrogen (NH_3_-N) was measured in the water extract as reported by Weatherburn (Weatherburn, 1967), using a spectrophotometer (8,500II, Thermo Electron Corporation, Waltham, MA, United States). The volatile fatty acid (VFA) content was analyzed as described previously by Kang et al. (2016).

### Assessment of the Feed Value

The relative feed value (RFV) was calculated from digestible dry matter digestibility (DMD) and dry matter intake (DMI) using ADF (%) and NDF (%), respectively (Rohweder et al., 1978). The RFV of silages was calculated as follow: DMI (%) = 120/NDF, DMD (%) = 88.9–0.779 × ADF, RFV = (DMI × DMD)/1.29.

### Analysis of Bacterial Community

The E.Z.N.A.^®^ Stool DNA Kit (D4015, Omega, Inc., United States) was utilized to extract the DNA from each sample, according to the instructions of the protocol for pathogen detection. The region V3–V4 of the bacterial 16S rRNA gene was amplified with A200 Gradient Thermal Cycler (Hangzhou LongGene Scientific Instruments Co., Ltd). The primers were as follow: 341F (CCTACGGGNGGCWGCAG) and 806R (GGACTACHVGGGTWTCTAAT) (Wang et al., 2019a). The reaction mixtures of PCR amplification were included template DNA (25 ng), PCR Premix (12.5 μl), and each primer (2.5 μl) in 25 μl total volume. The amplification conditions were as follows: an initial denaturation (98°C, 30 s); 35 cycles of denaturation (98°C, 10 s), annealing (54°C, 30 s), extension (72°C, 45 s); and extension (72°C, 10 min). Amplicon pyrosequencing was conducted on an Illumina MiSeq platform (LC-Biotechnology Co., Ltd., Hangzhou, China). Paired-end reads was assigned to samples based on their unique barcode and truncated by cutting off the barcode and primer sequence when the average quality score on the 10-bp sliding window was below 20. Reads that contained undetected nucleotides (N) or were shorter than 200 bp were removed. Paired-end reads with >10 bp of overlap were merged using FLASH software (version 1.2.8). Quality-filtering on the raw tags was performed under specific filtering conditions to obtain the high-quality clean tags according to the fqtrim (version 0.94). Vsearch software (version 2.3.4) was used to filter the chimeric sequences. Sequences (similarity > 97%) were assigned to the same operational taxonomic units (OTUs). The α-diversity analysis was calculated using mothur (version 1.36.0). The most abundant sequences in each OTU were used for taxonomic classification by Ribosomal Database Project (RDP) Classifier (version 11.5) with the confidence threshold as 80%. A principal coordinate analysis (PCoA) was completed via Muscle (version 3.8.31). The ANOSIM analysis was used to compare the differences between groups in PCoA analysis. Correlations between the main genera and silage quality were calculated *via* Spearman’s correlation analysis (SPSS, version 19.0, Chicago, IL, United States). All the sequences in the current study were deposited to the sequence read archive (SRA) of the NCBI database under the accession number PRJNA704787.

### Statistical Analysis

Two-way analysis of variance was utilized to evaluate the statistical significance. The fixed effects were the sucrose treatments, ensilage duration, and sucrose treatments by ensilage duration interactions, as calculated by the SPSS software (version 19.0, Chicago, IL, United States). All the data were presented as the means ± SEM. Tukey’s multiple comparison test was used to assess the differences between the means. Statistical significance was identified when *p* < 0.05, and a tendency was set at 0.05 < *p* < 0.10. GraphPad Prism software (Version 8.0, La Jolla, CA, United States) was used for all figures.

## Results

### Alfalfa Analysis Prior to Ensiling

Fresh alfalfa had a CP of 170 ± 2.5 g/kg DM, CF of 263 ± 8.0 g/kg DM, NDF of 460 ± 11.0 g/kg DM, ADF of 399 ± 21.5 g/kg DM, and a gross energy (GE) of 16.92 ± 0.1 MJ/kg. The DMI, DDM, and RFV values of the fresh alfalfa were 2.61 ± 0.06% body weight, 57.85 ± 1.68% DM, and 116.96 ± 0.60, respectively. The observed species, Shannon, Simpson, and Chao 1 indices for the fresh alfalfa were 410.33 ± 15.50, 3.65 ± 0.11, 0.80 ± 0.01, and 758.71 ± 74.54, respectively (Table 1).

**TABLE 1:** Alfalfa analysis prior to ensiling.

Item	Mean	SEM
***Chemical composition (g/kg DM)***		
CP	170	2.5
CF	263	8.0
NDF	460	11.0
ADF	399	21.5
GE (MJ/kg)	16.92	0.1
***Assessment of feed value***		
DMI/% BW	2.61	0.06
DDM/% DM	57.85	1.68
RFV	116.96	0.60
***Alpha diversity of the bacterial community***		
Observed species	410.33	15.50
Shannon	3.65	0.11
Simpson	0.80	0.01
Chao 1	758.71	74.54
Coverage	0.98	0.00

*CP, crude protein; CF, crude fiber; NDF, neutral detergent fiber; ADF, acid detergent fiber; GE, total energy; BW, body weight; DMI, dry matter intake; DDM, digestible dry matter; RFV, relative feed value; SEM, standard deviation of the mean. The data were calculated values based on estimating formulas.*

### Chemical Composition of the Alfalfa Silage

The DM of the alfalfa silage was significantly affected (*p* = 0.009) by the sucrose additions. The DM content was significantly increased (*p* < 0.05) in the AS2 group when compared with that in the AL group at 30 days and was comparable among the three groups at 15 days and 60 days. The CP and ADF content of the alfalfa silage was significantly affected (*p* < 0.05) by the sucrose additions, ensiling duration, and their interactions. The CP content at 60 days was significantly higher than that at 30 days (*p* < 0.05). The control silage always had the highest NDF and ADF content. Furthermore, the NDF and ADF content was comparable among the three groups at 15 days and significantly decreased (*p* < 0.05) in the AS2 silage at 30 and 60 days. The lowest NDF and ADF content was recorded in the AS2 silage at 60 days, with values of 399 and 261 g/kg DM, respectively. The CF content was significantly affected by the duration of ensiling (*p* < 0.05). After 60 days of ensiling, the minimum CF content was 215 g/kg DM in the AS2 silage. In addition, the interaction between the sucrose treatments and the ensiling durations significantly affected the GE values of the silages (*p* < 0.05). The GE values ranged from 17.00 to 17.80 MJ/kg among the three groups with the highest values at 60 days (Table 2).

**TABLE 2:** Chemical compositions of the alfalfa silages (g/kg DM).

Items	Treatment	Days ensiled			SEM	*p-*value		
		15	30	60		T	D	I
DM	AL	302	273^β^	290	9.02	**	ns	ns
	AS1	300	284^αβ^	287				
	AS2	313	309^α^	312				
CP	AL	177^b^	166^c^	188^a^	2.53	**	***	*
	AS1	180^a^	166^b^	182^a^				
	AS2	177^b^	172^b^	197^a^				
NDF	AL	409	418^α^	427^α^	8.83	*	ns	ns
	AS1	422	413^αβ^	419^αβ^				
	AS2	405	404^β^	399^β^				
ADF	AL	346^a^	324^αab^	304^αb^	7.96	***	***	*
	AS1	338	318^αβ^	317^αβ^				
	AS2	325^a^	316^βa^	261^βb^				
CF	AL	270^ab^	279^αa^	252^αb^	3.57	ns	***	ns
	AS1	264	265^αβ^	261^αb^				
	AS2	271^a^	257^βa^	215^βb^				
GE	AL	17.10^b^	17.19^b^	17.61^a^	0.11	ns	***	*
	AS1	17.15^b^	17.03^b^	17.80^a^				
	AS2	17.00	16.85	17.68				

*AL, 0% sucrose; AS1, 0.5% sucrose; AS2, 1.0% sucrose; DM, dry matter (g/kg fresh weight); CP, crude protein; NDF, neutral detergent fiber; ADF, acid detergent fiber; CF, crude fiber; GE, total energy (MJ/kg)T, treatments; D, ensiling time; I, interaction between ensiling duration and treatments; SEM, standard error of the mean.*

*^*a,b,c*^Within a row, means with different superscripts are different (*p* < 0.05).*

*^*α*,*β*,*γ*^ Within a column, means with different superscripts are different (*p* < 0.05).*

*p < 0.05; **p < 0.01; ***p < 0.001; and ns, not significant.

### Fermentation Dynamics of the Alfalfa Silage

The fermentation characteristics of alfalfa silages with or without sucrose are presented in Table 3. The interactions between the sucrose treatments and the ensiling durations significantly affected the pH value (*p* < 0.01). Compared with the AL silage, the 1% sucrose addition treatment significantly decreased (*p* < 0.05) the pH value for each duration. However, the pH values were comparable between the AL and AS1 groups at 30 and 60 days and significantly decreased (*p* < 0.05) in the AS1 silage at 15 days when compared with that of the AL silage.

**TABLE 3:** pH, NH_3__–_N, and VFA of the different alfalfa silages.

Items	Treatment	Days ensiled			SEM	*p-*Value		
		15	30	60		T	D	I
pH	AL	5.52^α^	5.45^α^	5.37^α^	0.078	***	ns	**
	AS1	5.15^βab^	5.49^αa^	4.96^αβb^				
	AS2	4.90^β^	4.75^β^	4.95^β^				
NH_3__–_N (g/kg TN)	AL	30.05^b^	40.25^αa^	42.23^αa^	1.71	*	***	ns
	AS1	32.42^b^	35.30^αβab^	41.45^αβa^				
	AS2	31.69^b^	33.01^βab^	37.68^βa^				
**Concentration of VFA (g/kg DM)**								
Acetic acid	AL	22.91^αb^	35.11^αb^	49.44^αa^	0.945	***	***	***
	AS1	17.09^βb^	22.24^βa^	23.23^βa^				
	AS2	15.43^βb^	15.95^γb^	25.47^βa^				
Propionic acid	AL	1.46^αc^	8.20^αb^	10.86^αa^	0.103	***	***	***
	AS1	1.21^αc^	3.57^βa^	2.44^βb^				
	AS2	0.50^βc^	1.03^γb^	2.11^βa^				
Butyric acid	AL	6.95^αb^	24.62^αa^	22.07^αa^	0.506	***	***	***
	AS1	5.23^αc^	22.93^αa^	16.72^βb^				
	AS2	1.90^βb^	13.05^βa^	13.78^γa^				

*AL, 0% sucrose; AS1, 0.5% sucrose; AS2, 1.0% sucrose; DM, dry matter; NH_3__–_N, ammonia nitrogen; TN, total nitrogen; T, treatments; D, ensiling duration; I, interaction between ensiling duration and treatments; SEM, standard error of means; VFA, volatile fatty acid.*

*^*a,b,c*^Means within a row with different superscripts differ (*p* < 0.05).*

*^*α*,*β*,*γ*^ Means within a column with different superscripts differ (*p* < 0.05).*

*p < 0.05; **p < 0.01; ***p < 0.001; and ns, not significant.

The NH_3_-N content was significantly affected (*p* < 0.05) by the sucrose additions and ensiling durations, and highest at terminal silage in AL, AS1, and AS2 silages. The NH_3_-N content was comparable among three groups at early phase of ensiling (*p* > 0.05). However, the 1.0% sucrose additions significantly decreased (*p* < 0.05) the NH_3_-N content compared with the AL silage at 30 and 60 days ensiling.

The contents of the acetic acid, propionic acid, and butyric acid in the alfalfa silage were significantly affected (*p* < 0.05) by the sucrose additions, ensiling durations, and their interactions. Compared with the AL silage, the 1% sucrose addition significantly decreased (*p* < 0.05) the acetic acid, propionic acid, and butyric acid content for each ensiling duration. In particular, the concentration of butyric acid was the lowest at 60 days.

### Assessment of the Feed Value

Sucrose additions significantly affected (*p* < 0.05) the DMI values, which were comparable among three groups at 15 days and significantly increased (*p* < 0.05) in the AS2 silage at 30 and 60 days. The DDM value of the alfalfa silage was significantly affected (*p* < 0.05) by the sucrose additions, ensiling durations, and their interactions. In addition, the DDM value was comparable among the three groups at 15 days and significantly increased (*p* < 0.05) in the AS2 silage at 30 and 60 days when compared with that in the AL silage. Furthermore, the DDM value reached its greatest value (66.28% DM) in the AS2 silage at 60 days and did not change in the AL and AS1 groups with the increasing ensiling durations. The RFV value of the silage was significantly affected (*p* < 0.05) by the sucrose treatments and the interactions between the sucrose treatments and ensiling durations. The RFV values were comparable among the three groups at 15 days and increased (*p* < 0.05) in the AS2 silage at 30 and 60 days, when compared with the AL and AS1 silages, with the greatest value being 160.04 (Table 4).

**TABLE 4:** Relative feed value of the alfalfa silage.

Items	Treatment	Days ensiled			SEM	*p-*Value		
		15	30	60		T	D	I
DMI/%	AL	2.97^a^	2.80^βab^	2.69^βb^	0.062	*	ns	ns
	AS1	2.85	2.91^αβ^	2.93^β^				
	AS2	2.97	2.97^α^	2.99^α^				
DDM/%	AL	60.81	63.05^β^	64.04^β^	0.620	***	***	*
	AS1	62.54	64.13^αβ^	66.26^αβ^				
	AS2	63.61^b^	64.30^αb^	66.28^αa^				
RFV	AL	140.06	137.10^γ^	133.86^β^	3.755	**	ns	*
	AS1	138.21	144.57^β^	150.80^β^				
	AS2	146.37	148.13^α^	160.04^α^				

*AL, 0% sucrose; AS1, 0.5% sucrose; AS2, 1.0% sucrose; DMI, dry matter intake; DDM, digestible dry matter; RFV, relative feed value; T, treatments; D, ensiling duration; I, interaction between ensiling duration and treatments; SEM, standard error of the mean.*

*The data were calculated based on the estimating formulas.*

*^*a,b,c*^Means within a row with different superscripts differ (*p* < 0.05).*

*^*α*,*β*,*γ*^ Means within a column with different superscripts differ (*p* < 0.05).*

*p < 0.05; **p < 0.01; ***p < 0.001; ns, not significant.

### Bacterial Community Diversity in the Alfalfa Silage

Bacterial diversity analysis of alfalfa silage ensiling day 15, 30, and 60 are presented in Table 5. The ensiling durations and the interactions between the sucrose treatments and ensiling durations significantly affected (*p* < 0.05) the OTUs, observed species, and Chao 1 indices of the alfalfa silage. The OTUs reached their highest value in the AS1 silage at 60 days. Furthermore, there was a U-shaped curvilinear relationship between the OTUs, Chao indices, and sucrose additions at 30 days, although they were comparable in the OTU and Chao indices between the AL and AS2 groups. In addition, the Shannon and Simpson indices were influenced (*p* < 0.01) by the ensiling duration. Moreover, the Simpson index reached its highest value in the AL group after 60 days of ensiling. However, the Shannon and Simpson indices were comparable among the three groups for each ensiling duration.

**TABLE 5:** Alpha diversity of the alfalfa silage bacterial communities.

Items	Treatment	Days ensiled			SEM	*p-*Value		
		15	30	60		T	D	I
OTU_S_	AL	622	575^αβ^	675^γ^	48.13	ns	***	**
	AS1	633^b^	449^βγb^	956^αa^				
	AS2	540^b^	700^αab^	776^βa^				
Observed species	AL	493^α^	487^αβ^	533	23.83	ns	**	***
	AS1	491^αbc^	390^βγc^	546^ab^				
	AS2	380^βb^	543^αa^	505^a^				
Chao 1	AL	863	784^αβ^	949	55.01	ns	*	*
	AS1	775^bc^	672^βγc^	920^ab^				
	AS2	689	905^α^	853				
Shannon	AL	4.35^bc^	4.31^c^	5.11^ab^	0.192	ns	**	ns
	AS1	4.38	4.12	4.84				
	AS2	4.08	4.79	4.59				
Simpson	AL	0.84^b^	0.81^b^	0.91^a^	0.026	ns	**	ns
	AS1	0.81	0.79	0.89				
	AS2	0.80	0.88	0.87				
Coverage	AL	0.98	0.98	0.98	–	–	–	–
	AS1	0.98	0.98	0.98				
	AS2	0.98	0.98	0.98				

*AL, 0% sucrose; AS1, 0.5% sucrose; AS2, 1.0% sucrose; T, treatments; D, ensiling duration; I, interaction between ensiling duration and treatments; SEM, standard error of means.*

*The data were calculated based on estimating formulas.*

*^*a,b,c*^Means within a row with different superscripts differ (*p* < 0.05).*

*^*α*,*β*,*γ*^ Means within a column with different superscripts differ (*p* < 0.05).*

*p < 0.05; **p < 0.01; ***p < 0.001; and ns, not significant.

**FIGURE 1:**
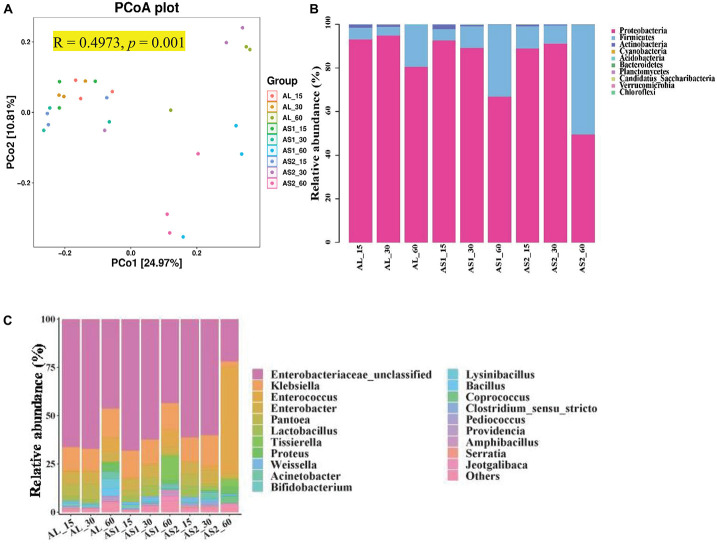
**(A)** Principal coordinate analysis (PCoA). **(B)** Relative abundance of microbiota at the level of phylum in the alfalfa silage samples. **(C)** Relative abundance of microbiota at the level of genus.

Principal coordinates analysis (PCoA) which was UniFrac-based showed a distinct clustering of the microbiota compositions for each group (*R* = 0.4973, *p* = 0.001) (Figure 1A). Principle coordinates 1 and 2 accounted for 22.97% and 10.81% of the total variance, respectively (Figure 1A). Taxonomic analysis showed that the microbiota of the alfalfa silage was represented by two major phyla: *Proteobacteria* and *Firmicutes* (Figure 1B). The relative abundance of these two major phyla was significantly affected (*p* < 0.001) by the sucrose addition, ensiling duration, and their interactions. Moreover, the relative abundance of *Proteobacteria* reached its lowest value and that of *Firmicutes* reached its highest value in the AS2 group after 60 days of ensiling (Table 6).

**TABLE 6:** Relative abundance of the major phyla (%) of the alfalfa silage.

Items	Treatment	Days ensiled			SEM	*p-*Value		
		15	30	60		T	D	I
*Proteobacteria*	AL	93.06	94.87	80.51^α^	2.28	***	***	***
	AS1	92.57	89.14	80.87^α^				
	AS2	88.88^a^	91.11^a^	32.89^βb^				
*Firmicutes*	AL	5.31^ab^	4.00^b^	19.41^βa^	1.95	***	***	***
	AS1	5.23	9.94	18.88^β^				
	AS2	10.21^b^	8.35^b^	66.78^αa^				

*AL, 0% sucrose; AS1, 0.5% sucrose; AS2, 1.0% sucrose; T, treatments; D, ensiling duration; I, interaction between ensiling duration and treatments; SEM, standard error of means.*

*The data were calculated based on estimating formulas.*

*^*a,b,c*^Means within a row with different superscripts differ (*p* < 0.05).*

*^*α*,*β*,*γ*^ Means within a column with different superscripts differ (*p* < 0.05).*

***p < 0.001.

At the genus level, *Enterobacteriaceae*, *Klebsiella*, and *Enterococcus* were dominant in the alfalfa silages, followed by *Enterobacter*, *Pantoea, Lactobacillus*, *Proteus*, *Acinetobacter*, and *Bifidobacterium* (Figure 1C). The relative abundances of *Enterobacteriaceae*, *Enterococcus*, *Pantoea*, and *Proteus* were significantly affected (*p* < 0.05) by the sucrose addition, ensiling duration, and their interactions. The relative abundance of *Klebsiella* was significantly affected (*p* < 0.01) by the sucrose addition, the interaction of sucrose addition, and ensiling duration. The relative abundances of *Enterobacter, Lactobacillus*, and *Bifidobacterium* were significantly affected (*p* < 0.01) by the ensiling duration. Furthermore, the relative abundances of *Enterobacteriaceae*, and *Klebsiella* were significantly decreased (*p* < 0.05) and that of *Enterococcus and Lactobacillus* were significantly increased (*p* < 0.05) in the AS2 group at 60 days when compared with that in the AL group or the AS1 group. In addition, the relative abundance of *Pantoea* in the AS2 group was lower than that in the AS1 group at 60 days (Table 7).

**TABLE 7:** Relative abundance of genera (%) of the alfalfa silage.

Items	Treatment	Days ensiled			SEM	*p-*Value		
		15	30	60		T	D	I
*Enterobacteriaceae*	AL	66.24^a^	67.08^a^	46.22^αb^	3.22	***	***	**
	AS1	68.15^a^	62.43^ab^	55.24^αb^				
	AS2	61.18^a^	60.06^a^	21.83^βb^				
*Klebsiella*	AL	12.37	11.41	15.00^αβ^	1.25	**	ns	***
	AS1	13.92^b^	12.38^b^	19.95^αa^				
	AS2	12.52^a^	15.65^a^	2.88^βb^				
*Enterococcus*	AL	0.46^ab^	0.36^b^	7.38^βa^	0.06	***	***	***
	AS1	0.49^b^	0.52^b^	6.01^βa^				
	AS2	0.51^b^	2.63^b^	55.19^αa^				
*Enterobacter*	AL	6.43^a^	6.49^a^	4.93^b^	0.12	ns	***	ns
	AS1	6.04^a^	6.47^a^	4.36^b^				
	AS2	6.00^a^	6.88^a^	2.56^b^				
*Pantoea*	AL	6.13^a^	8.03^αa^	0.56^αβb^	0.08	*	***	**
	AS1	3.24^ab^	4.48^αβa^	0.89^αb^				
	AS2	6.27^a^	1.53^βab^	0.28^βb^				
*Lactobacillus*	AL	5.28	2.05	0.03^β^	0.001	ns	**	ns
	AS1	2.89^ab^	5.60^a^	0.04^βb^				
	AS2	5.67	1.87	0.39^α^				
*Proteus*	AL	0.00^b^	0.00^b^	4.71^αa^	0.01	***	***	***
	AS1	0.00^b^	0.00^b^	1.08^βa^				
	AS2	0.00^b^	0.81^b^	3.46^αa^				
*Acinetobacter*	AL	0.06^b^	0.08^βb^	3.77^a^	0.02	ns	ns	ns
	AS1	0.04^b^	0.31^αβb^	1.89^a^				
	AS2	0.12	3.03^α^	0.07				
*Bifidobacterium*	AL	1.05	0.98	0.00^β^	0.01	ns	**	ns
	AS1	2.04^a^	0.51^ab^	0.03^αβb^				
	AS2	0.69	0.36	0.11^α^				

*AL, 0% sucrose; AS1, 0.5% sucrose; AS2, 1.0% sucrose; T, treatments; D, ensiling duration; I, interaction between ensiling duration and treatments; SEM, standard error of means.*

*The data were calculated based on estimating formulas.*

*^*a,b,c*^Means within a row with different superscripts differ (*p* < 0.05).*

*^*α*,*β*,*γ*^ Means within a column with different superscripts differ (*p* < 0.05).*

*p < 0.05; **p < 0.01; ***p < 0.001; and ns, not significant.

### Relationship Between Main Genera and Silage Quality

**FIGURE 2:**
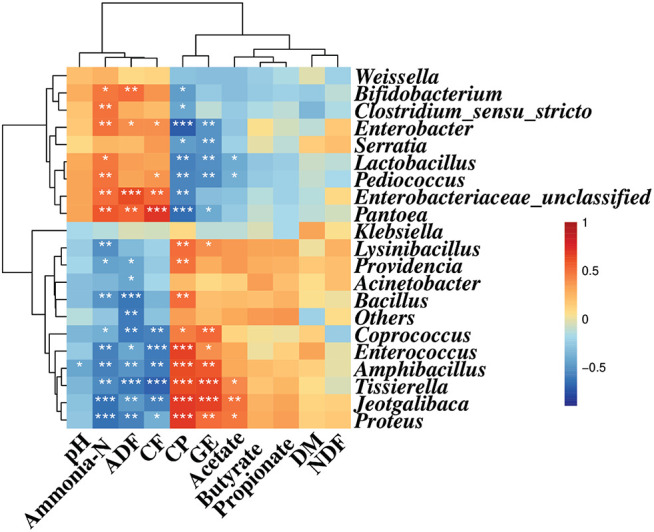
Spearman correlation heatmap between the main genera and silage quality. R was presented in different colors; the right side of the legend is the color range of different R values. AL, control; AS1, 0.5% sucrose; AS2, 1.0% sucrose. The value of *p* ≤ 0.05 is marked with “*”, *p* ≤ 0.01 is marked with “**”, and *p* ≤ 0.001 is marked with “***”.

Spearman correlation analysis illustrated that *Enterobacter*, *Enterobacteriaceae*, and *Pantoea* were positively correlated with ammonia-N (*r* = 0.49, 0.51, and 0.58, respectively, *p* < 0.05), ADF (*r* = 0.39, 0.63, and 0.55, respectively, *p* < 0.05), and CF (*r* = 0.40, 0.54, and 0.70, respectively, *p* < 0.05) contents, while they were negatively correlated with the CP content (*r* = −0.71, −0.57, and −0.66, respectively, *p* < 0.05). Also, *Bifidobacterium*, *Clostridium*, *Serratia*, *Lactobacillus*, and *Pediococcus* were negatively correlated with CP (*r* = −0.47, −0.43, −0.46, −0.58, and −0.57, respectively, *p* < 0.05) content. Meanwhile, *Bifidobacterium*, *Clostridium*, *Lactobacillus*, and *Pediococcus* were positively correlated with the concentration of ammonia-N (*r* = 0.48, 0.50, 0.47, and 0.50, respectively, *p* < 0.05) and CF (*r* = 0.37, 0.23, 0.31, and 0.38, respectively, *p* < 0.05) contents, and *Bifidobacterium* was positively correlated with the ADF content (*r* = 0.52, *p* < 0.05) (Figure 2).

*Coprococcus*, *Enterococcus*, *Amphibacillus*, *Tissierella*, *Jeotgalibaca*, and *Proteus* displayed positive correlation with CP content (*r* = 0.45, 0.67, 0.62, 0.62, 0.69, and 0.69, respectively, *p* < 0.05) and GE value (*r* = 0.54, 0.40, 0.60, 0.65, 0.66, and 0.51, respectively, *p* < 0.05). However, they showed negative correlation with ammonia-N (*r* = −0.39, −0.58, −0.59, −0.64, and −0.64, respectively, *p* < 0.05), ADF (*r* = −0.59, −0.42, −0.50, −0.62, −0.49, and −0.58, respectively, *p* < 0.05), and CF (*r* = −0.59, −0.61, −0.60, −0.70, −0.58, and −0.40, respectively, *p* < 0.05) contents. Likewise, *Lysinibacillus*, *Bacillus*, and *Providencia* were positively correlated with the CP content (*r* = 0.52, 0.51, and 0.49, respectively, *p* < 0.05) and negatively correlated with ammonia-N content (*r* = −0.52, −0.50, and −0.46, respectively, *p* < 0.05). The ADF content was also negatively correlated with *Providencia* and *Acinetobacter* (*r* = −0.43, −0.42, respectively, *p* < 0.05). The GE value was positively correlated with *Lysinibacillus* (*r* = 0.40, *p* < 0.05). In addition, the acetate concentration was negatively correlated with *Pediococcus* and *Lactobacillus* (*r* = −0.39, −0.41, respectively, *p* < 0.05), and positively correlated with *Proteus*, *Jeotgalibaca*, and *Tissierella* (*r* = 0.47, 0.50, and 0.41, respectively, *p* < 0.05) (Figure 2).

## Discussion

Silage pH is the key factor influencing the fermentation quality (Muck et al., 2018). In the AS2 groups, the pH value rapidly declined to a significantly lower level than the pH value in AL group at the early stage of fermentation and was stable with the prolonged ensiling time. Moreover, the production of butyric acid results in the loss of energy and butyric acid usually results in livestock reducing their intake. So, it is unwanted in silage (Muck, 2010). The significantly lower pH value and butyric acid content in the AS2 silage than in the AL silage in this study indicated that the growth of harmful bacteria was inhibited by the pH and that sucrose inclusion could improve the quality of the ensiling fermentation products. This was consistent with the results of a previous study that 0.67% sucrose addition decreased the pH value of alfalfa silage after 120 days of fermentation (Li et al., 2016). Relative feed value (RFV) is an evaluation criteria to express the forage quality. The more of the RFV, the better is the forage quality (Rohweder et al., 1978). The higher RFV values in the AS2 silage at 30 and 60 days fermentation indicated that 1% sucrose addition improved the feeding quality of alfalfa silage.

The ammonia-N content usually reflects the silage proteolysis during ensiling, and is commonly driven by plant enzyme protease and proteolytic *Clostridia* (Wang et al., 2019c). The lower ammonia-N content in AS2 at terminal silage indicated that the sucrose additions contributed to the protein preservation. This result was consistent with previous study that sucrose additions decreased ammonia-N content in alfalfa silage (Li et al., 2020). Moreover, the loss of DM is exacerbated by the acetic acid bacteria. Therefore, the acetic acid content is closely related to the loss of DM in silage (Guo et al., 2020). In this study, the lower acetic acid content after 60 days of ensiling in both the AS1 and AS2 silages suggested that sucrose additions might decrease the loss of DM in the silage. A similar observation has been reported previously, as 0.67% sucrose additions were found to decrease the acetic acid content of alfalfa silage after 120 days of fermentation (Li et al., 2016). Moreover, *Enterobacteriaceae* can enhance the production of ammonia-N (Queiroz et al., 2018), and acetic acid is mainly produced by the *Enterobacteriaceae* during the fermentation process (Wang et al., 2020). Therefore, the lower ammonia-N and acetic acid contents observed in the AS2 group coincided with the significantly lower *Enterobacteriaceae* populations in the AS2 group than AL group after 60 days of fermentation.

The coverage of microbial DNA samples in the present study was nearly 1, suggesting that most bacteria in the samples were represented by 16S rRNA sequences. Generally, the alpha diversity of the bacteria was estimated by using the number of OTUs, Chao1, and Shannon index. The ensiling time significantly affected α diversity at present study, indicating that the richness and evenness of bacterial community was influenced by the silage time. The similar Shannon and Chao1 indices among the three groups on days 15 and 60 silage in current study indicated that the richness and evenness of the bacterial communities was stable at initial and terminal silage when the sucrose was supplemented. The PCoA analysis in this study suggested that sucrose addition had an effect on microbial composition. This result was similar with the report that alfalfa had different bacterial population after ensiling, but the bacterial diversity was changed very little (McGarvey et al., 2013). The reason for this phenomenon might be that the changes in the level of some taxonomic groups are offset by opposite changes in other groups (Hartmann and Windmer, 2006). In addition, the silage time significantly affected α diversity at present study, indicating that the richness and evenness of bacterial community was influenced by the silage time.

After ensiling, *Proteobacteria* and *Firmicutes* became the dominant phyla, with or without sucrose additions. A previous investigation similarly showed that the most abundant phyla in mulberry silage were *Proteobacteria* and *Firmicutes*, with or without sucrose additions, after 60 days of ensiling (Wang et al., 2019b). The domination of these two phyla might be due to the low pH and anaerobic conditions of the silage, which were conducive to their growth. *Firmicutes* are vital acid hydrolytic microbes under anaerobic condition, which could produce numerous extracellular enzymes (Wang et al., 2020). The significantly lower *Proteobacteria* and higher *Firmicutes* populations in AS2 group than AL group after 60 days of fermentation indicated that the bacterial population structure of the alfalfa shifted significantly during the ensiling process when the 1% sucrose was added. The reason for this result was that the acidic and anaerobic environment during ensiling is beneficial for the proliferation of *Firmicutes* (Keshri et al., 2018). This result was in accordance with previous report that the amount of *Proteobacteria* was declined, while the numbers of *Firmicutes* was markedly increased in alfalfa silage (McGarvey et al., 2013).

The genera *Enterococcus* and *Lactobacillus* are known to dominate the fermentation of forage products when anaerobic conditions are formed. Furthermore, the silage quality was improved by using these genera (Zhang et al., 2017). Moreover, *Lactobacillus* and *Enterococcus* belong to the lactic acid bacteria, and they initiate lactic fermentation at the early ensiling process, while *Enterococcus* had lower tolerance to low pH than *Lactobacillus* (Bai et al., 2021). However, *Enterobacteriaceae*, *Klebsiella*, and *Enterococcus* were the dominant genera in the current study, which were different from the previous observed microflora in alfalfa silages (McGarvey et al., 2013; Guo et al., 2018). Moreover, we observed a higher relative abundance of *Enterococcus* in the AS2 silage in the present study. *Enterococcus* is often applied to enhance the fermentation characteristics and play a pivotal role in accelerating the lactic acid fermentation and building an anaerobic acidic circumstance for the development of *Lactobacillus* (Cai et al., 1998). The probable reason for this result was the slow rate of pH decline in AS2 silages.

*Lactobacillus* plays an important role in pH reduction during the later stages of lactic fermentation (da Silva et al., 2020). Although *Lactobacillus* was not the dominant genera in the AS2 silage in this study, the relative abundance of *Lactobacillus* in the AS2 silage was greater than in the AL silage in the current study. This indicated that sucrose addition can promote the fermentation quality of alfalfa silage. Similarly, the alfalfa silages were relatively abundant in lactic acid bacteria when treated with *E. coli* O157:H7 and *Lactobacillus plantarum* (Ogunade et al., 2018).

The results shown in Figure 2 indicated that there was an interaction between the bacterial community and silage fermentation. The negative correlation among *Coprococcus*, *Enterococcus*, *Amphibacillus*, *Tissierella*, *Jeotgalibaca*, *Proteus*, and ammonia-N concentration in the present study indicated their contribution to protein preservation.

## Conclusion

The ensiling time significantly decreased the crude fiber content, and increased the ammonia nitrogen, acetic acid content, and the relative abundance of *Enterococcus* in the silages. Supplementing alfalfa silage samples with different doses of sucrose decreased their pH levels and NDF, propionic acid, and butyric acid content. There was an increment in the RFV levels with the increments of the sucrose supplementations. Furthermore, the reduced levels of butyric acid suppressed harmful bacteria and the relative abundance of *Lactobacillus* and *Enterococcus* in the silage increased. In conclusion, our results suggest that sucrose supplementation improves the feeding quality of alfalfa silage.

## Data Availability Statement

The datasets presented in this study can be found in online repositories. The names of the repository/repositories and accession number(s) can be found below: https://www.ncbi.nlm.nih.gov/sra/PRJNA704787, PRJNA704787.

## Author Contributions

JK handled the validation, investigation, methodology, visualization, writing of the original draft, and reviewing and editing of the manuscript. ST contributed to the validation, formal analysis, reviewing, editing, and writing of the draft. RZ contributed to the validation, reviewing, editing, and writing of the draft. ZT contributed to the validation, writing, reviewing, editing of the manuscript, and supervision. DW participated in the validation, conceptualization, supervision, writing, reviewing, editing of the manuscript, and was in charge of the funding acquisition and project administration. All authors contributed to the article and approved the submitted version.

## Conflict of Interest

The authors declare that the research was conducted in the absence of any commercial or financial relationships that could be construed as a potential conflict of interest.

## Publisher’s Note

All claims expressed in this article are solely those of the authors and do not necessarily represent those of their affiliated organizations, or those of the publisher, the editors and the reviewers. Any product that may be evaluated in this article, or claim that may be made by its manufacturer, is not guaranteed or endorsed by the publisher.
